# Acupuncture modulates the frequency-specific functional connectivity density in primary dysmenorrhea

**DOI:** 10.3389/fnins.2022.917721

**Published:** 2022-08-16

**Authors:** Li-Ying Liu, Xiang Li, Zi-Lei Tian, Qi Zhang, Zhi-Fu Shen, Wei Wei, Xiao-Li Guo, Ling Chen, Meng-Hua Su, Lu Yang, Si-Yi Yu, Jie Yang

**Affiliations:** ^1^Acupuncture and Tuina School, Chengdu University of Traditional Chinese Medicine, Chengdu, China; ^2^Chongqing Hospital of Traditional Chinese Medicine, Chongqing, China; ^3^Department of Traditional Chinese and Western Medicine, North Sichuan Medical College, Nanchong, China; ^4^Chengdu Xinan Gynecological Hospital, Chengdu, China

**Keywords:** primary dysmenorrhea, acupuncture, resting-state fMRI, functional connectivity density, frequency band

## Abstract

**Background:**

The study aimed to investigate how acupuncture modulates brain activities across multiple frequency bands to achieve therapeutic effects in PDM.

**Methods:**

A total of 47 patients with PDM were randomly assigned to the verum acupuncture group and sham acupuncture group with three menstrual cycles of the acupuncture course. The fMRI scans, visual analog scale (VAS) scores, and other clinical evaluations were assessed at baseline and after three menstrual-cycles treatments. The global functional connectivity density (gFCD) analyses were performed between the pre-and post-acupuncture course of two groups at full-low frequency band, Slow-3 band, Slow-4 band, and Slow-5 band.

**Results:**

After the acupuncture treatments, the patients with PDM in the verum acupuncture group showed significantly decreased VAS scores (*p* < 0.05). The frequency-dependent gFCD alternations were found in the verum acupuncture group, altered regions including DLPFC, somatosensory cortex, anterior cingulate cortex (ACC), middle cingulate cortex (MCC), precuneus, hippocampus, and insula. The sham acupuncture modulated regions including angular gyrus, inferior frontal gyrus, and hippocampus. The gFCD alternation in DLPFC at the Slow-5 band was negatively in the patients with PDM following verum acupuncture, and S2 at the Slow-4 band was positively correlated with VAS scores.

**Conclusion:**

These findings supported that verum acupuncture could effectively modulate frequency-dependent gFCD in PDM by influencing abnormal DLPFC at Slow-5 band and hippocampus at the Slow-3 band. The outcome of this study may shed light on enhancing the potency of acupuncture in clinical practice.

## Introduction

Primary dysmenorrhea (PDM), characterized by persistent menstrual discomfort in the absence of pelvic abnormalities, is a common gynecological condition affecting between 17 and 90% of females of reproductive age. However, the pathogenesis of PDM is not fully understood. Recently, neuroimaging studies showed that the prolonged PDM alternates brain structure and function, providing a new viewpoint on PDM diagnosis and treatment.

Resting-state functional magnetic resonance imaging (rs-fMRI) is a non-invasive imaging technique that provides a reliable representation of the brain’s spontaneous functional activity (Fox and Raichle, 2007; Baliki et al., 2011a). It offers new insights into the underlying spontaneous brain activity associated with chronic pain. The previous studies identified the activation of primary (S1) and second somatosensory cortex (S2), anterior cingulate cortex (ACC), insula (INS), dorsolateral prefrontal cortex (DLPFC), and hippocampus in patients with chronic pain (Liu and Chen, 2009; Fomberstein et al., 2013; Schmidt-Wilcke, 2015; Schmidt-Wilcke and Diers, 2017). Furthermore, the frequency-dependent alternations of brain activities in chronic pain were also demonstrated (Baliki et al., 2011a; Kilpatrick et al., 2014; Alshelh et al., 2016).

With the global functional connectivity density (gFCD) analysis, a graph-based and data-driven approach that quantifies the number of whole-brain connections over the whole-brain (Tomasi et al., 2016), our previous study first confirmed that the patients with PDM exhibited frequency-dependent distribution patterns of brain activity. Specifically, these altered brain areas, especially in the central executive network (CEN), default mode network (DMN), sensorimotor network (SMN), and the hippocampus (Yu et al., 2021). Notably, we observed a decreased gFCD in DLPFC at the Slow-5 band and an increased gFCD in the hippocampus at the Slow-3 band, indicating that the patients with PDM showed aberrant pain processing. As a result, we confirmed that the patients with PDM showed distinct functional brain activity frequency specific.

Acupuncture is a non-pharmacological intervention for analgesia that has been recognized (Berman et al., 2004; Witt et al., 2008; Hinman et al., 2014; Hershman et al., 2018). Over the last two decades, fMRI has been utilized to investigate the underlying brain mechanism of acupuncture, and studies have revealed that acupuncture significantly modulates cortical/subcortical brain areas involved in pain processing, cognition, and emotional processing (Bai and Lao, 2013; Cai et al., 2018; Zhang et al., 2020). Consequently, it is a reliable method to use fMRI to reveal the imaging mechanism of acupuncture for PDM.

In this study, we hypothesized that acupuncture might normalize the brain regions previously identified (Yu et al., 2021) as aberrant in PDM for therapeutic purposes, and this modulation was of frequency specific. First, we investigated how acupuncture modulates brain activity by analyzing gFCD changes across multiple frequency bands before and after treatment. Second, we evaluated the differences in brain regions modulated by verum acupuncture (VA) vs. sham acupuncture (SA) to determine if modulation of acupuncture on brain activity in PDM is a therapeutic effect. In addition, we performed a correlation analysis to determine the relationship between acupuncture-induced gFCD changes and clinical results.

## Material and methods

### Study design

This was a two-arm, randomized rs-fMRI clinical trial. The patients with PDM were randomized to VA and SA groups using a random number table. The patients with PDM were required to fulfill the dysmenorrhea diary to characterize their PDM symptoms in altogether 6 months.

### Participants

The inclusion and exclusion criteria in this study were similar to our previous study (Yu et al., 2021). The patients with PDM were recruited primarily through the gynecology outpatient and inpatient sections at the Affiliated Hospital of Chengdu University of Traditional Chinese Medicine, Chengdu University of Traditional Chinese Medicine, Sichuan University, Southwest University of Finance and Economics, Southwest Jiaotong University, and Southwest University for Nationalities. The recruitment period was from December 2015 to July 2018. Well-informed consent was obtained from all involved participants. The study protocol was approved by the medical ethics review of the Sichuan Regional Ethics Review Committee of Traditional Chinese Medicine (No. 2013KL-033).

The patients with PDM were enrolled fulfilling the following inclusion criteria: (1) Women aged 18–30 years, right-handed; (2) those with PDM diagnosed using the Society of Obstetricians and Gynecologists of Canada’s diagnostic criteria (Lefebvre et al., 2005); (3) patients with regular menstrual cycle (27–32 days); (4) patients with at least 1-year history of PDM; and (5) no exogenous hormones or centrally acting medicine in the previous 6 months; (6) pain assessed by visual analog scale (VAS) averaged over four points in the last 3 months. The exclusion criteria were as follows: Patients with (1) diagnosis of secondary dysmenorrhea due to organic pathology by ultrasound or gynecological examination; (2) suffering from other chronic pain conditions; (3) severe basic disorders; (4) mental illness, or with severe psychiatric disorders; (5) pregnant, preparing for pregnancy or breastfeeding; (6) treated with acupuncture in the past 3 months or have been taking analgesic medication for a long period; (7) contraindications to MRI examination; and (8) severe cranial anatomical asymmetry or well-defined lesions found on MRI scans.

### Acupuncture manipulations

Acupuncture treatments were performed by qualified practitioners who had undergone extensive clinical and operational training. The selection of the VA and SA was identical to the previous study (Wang et al., 2021). The VA acupoints were bilateral SP6, which were known to alleviate PDM well (Abaraogu et al., 2016; Yu et al., 2017). The SA acupoints were located bilaterally at the midpoint between SP6 and the Bladder meridian without known clinical effects. The location of the acupoints is shown in Supplementary Figure 1. The patients with PDM in the VA group were required to obtain deqi sensation (a complex phenomenon including soreness, numbness, heaviness, swelling, or dull pain achieved by pricking the needle 5–15 mm subcutaneously and gently manipulating) (Kong et al., 2007). The duration of the VA or SA was 30 min, once a day, during which the needles were retained without any manipulation. All patients received three menstrual cycles of acupuncture treatment, beginning 5–7 days before menstruation and ending until the onset of menstruation.

### Clinical outcomes

All clinical outcomes were assessed at Month 0 (baseline) and Month 3 (post-treatment). The primary outcome was VAS (McCormack et al., 1988) scores, in which the score from 0 to 10 indicates the pain level from low to high. In addition, Cox Menstrual Symptom Scale (CMSS) (Cox and Meyer, 1978) was utilized to assess the severity (CMSS-s) and time (CMSS-t) of PDM symptoms on 18 items. To assess anxiety and depressive symptoms, the Zung Self-Rating Anxiety Scale (SAS) (Zung, 1971) and the Zung Self-Rating Depression Scale (SDS) (Zung et al., 1965) were employed. Noteworthy, retrospective scoring (VAS, CMSS, SAS, and SDS) of 3 months before the enrollment was used as the baseline.

### Functional magnetic resonance imaging acquisition

This study used a 3.0-tesla magnetic resonance scanner (Discovery MR750, General Electric, Milwaukee, WI, United States) to perform scans. The participants were instructed to keep their eyes closed and awake during the scan. All subjects underwent MRI scans within 72 h of menstruation, with a total of two scans (including three scans in the baseline period and three scans in the treatment period).

First, a conventional three-plane localization was performed. The T1-weighted fast spoiled gradient–echo sequence was applied with the following parameters: Repetition time (TR) = 2.53 ms, echo time (TE) = 3.39 ms, field of view (FOV) = 256 mm × 256 mm, flip angle = 7°, slice thickness = 1 mm, and resolution = 256 × 256 × 188. The gradient–echo T2-weighted echo–planar imaging sequence was applied for resting-state fMRI, with parameters: TR = 2,000 ms, TE = 30 ms, FOV = 240 mm × 240 mm, flip angle = 90^°^, intra-layer resolution = 64 × 64, layer thickness = 4 mm, and number of slices = 32. Totally 250 scan time point were obtained.

### Data analysis

#### Clinical outcomes

Statistics analyses were performed using the SPSS 20.0 statistical software (SPSS Inc., Chicago, IL). Comparison of baseline characteristics (continuous variables) between the VA and SA group was statistically performed by the independent sample *t*-test. The variations of clinical outcomes (VAS scores, CMSS-t scores, CMSS-s scores, SAS scores, and SDS scores) before and after the treatment in the two groups were statistically determined by paired samples *t*-test. A repeated measurement of analysis of variance (RMANOVA) was performed on the values of changes in clinical outcomes before and after VA compared to SA. The significance level for statistical analysis of two-tailed testing was *p* < 0.05. The Pearson correlation analysis was applied to correlate the changes in VAS scores, CMSS-t scores, CMSS-s scores, SAS scores, and SDS scores before and after the treatment within the group. The significance level for statistical analysis of two-tailed testing was *p* < 0.05.

#### Functional magnetic resonance imaging data preprocessing

The data preprocessing for resting-state fMRI was performed by the Statistical Parametric Mapping (SPM12)^1^ in MATLAB 2014a (Mathworks, Inc., Natick, MA, United States). The first 10-time points were discarded, slice-timing correction, head motion estimation, normalization to standard Montreal Neurological Institute (MNI) EPI template and spatial smoothing with a 6-mm, and full-width-at-half-maximum Gaussian kernel were performed for the remaining 240-time points. Nuisance covariates regression was applied including six-direction head motion parameters, white matter, and cerebrospinal fluid (Ciric et al., 2017; Tu et al., 2019). The full low frequency (FLF) of 0.01–0.08 Hz was performed for functional connectivity analysis. Based on the study of Rogachov et al. (2018) and our previous study (Yu et al., 2021) on frequency-related neuroimaging studies of chronic pain, three different frequency band-based filters were selected for analysis, including Slow-5 band (0.01–0.027 HZ), Slow-4 band (0.027–0.073 Hz), and Slow-3 band (0.073–0.198 Hz).

#### Global functional connectivity density calculation

The FCD was calculated by the BRANT toolkit^2^ in MATLAB 2014a. The FCD of each voxel was calculated according to the method described by Tomasi and Volkow (Tomasi and Volkow, 2010, 2011, 2014). The gFCD value for a given voxel is the total number of active functional connections possessed by the voxel. Fisher *Z*-transformed version of correlation coefficient was the normalization method for FCD matrix. Pearson linear correlation analysis was performed to calculate the linear correlation between a given voxel (*i*) and all other voxels in the whole-brain as the number of global functional connections *k* (*i*), at a given voxel (*i*). Voxel pairs with a correlation coefficient of *r*_0_ > 0.6 were considered a significant connection. The gFCD calculations were limited to the cerebral gray matter mask (*N*_*voxels*_) region, setting a signal-to-noise ratio greater than 50% to minimize the adverse effects of signal loss and artifacts associated with magnetic sensitivity (Tomasi and Volkow, 2010).

Group analysis was applied using a random-effects model at different frequency bands. First, a voxel-based paired *t*-test was performed to measure the change in gFCD before and after the treatment in the VA or SA groups. Second, the brain regions that decreased or increased significantly after the treatment in the VA group compared with the SA group were explored by RMANOVA. Age was considered as a covariate in the statistics. For brain regions explicitly associated with pain in the previous studies that could not be corrected by family-wise error (FWE), a small-volume (anatomical structure) correction based 3dClustSim was taken by AFNI version 18.0.25 (Worsley et al., 1996).^3^ The threshold of voxel-wise *p* < 0.005 and *p* < 0.05 FWE corrected at cluster level (more than 20 consecutive voxels) was applied for all the analyses.

#### Correlation analysis

We conducted a Pearson linear correlation analysis to explore the clinical relevance of gFCD changes in brain regions identified in the previous study (Yu et al., 2021) by extracting the average *Z*-score values of the significantly altered gFCD clusters. The SPSS 20.0 statistical software was used to conduct the analysis.

## Results

A total of 58 patients with PDM were recruited in the study. Among the 47 patients with PDM who completed baseline clinical observations and fMRI scans, 41 patients (22 in the VA group, 19 in the SA group) completed 3-month treatment. Seven patients were excluded from the data processing for incomplete fMRI data (1 in the SA group and 2 in the VA group) and head movement exceeding 1.5 mm (1 in the VA group and 3 in the SA group). The flow chart was shown in Supplementary Figure 2.

### Clinical outcomes

#### Baseline characteristics

Table 1 showed the baseline and clinical characteristics in the statistics. Twenty patients with PDM (aged 24.70 ± 2.11 years) in the VA group and 14 patients with PDM (aged 24.29 ± 1.90 years) in the SA group were ultimately included in the statistical analysis. Of note, a moderate menstrual pain was experienced with VAS scores of 6.18 ± 0.99 in the VA group, and 5.93 ± 1.21 in the SA group. There was no significant difference in age, duration of PDM, and the height and weight between the two groups (*p* > 0.05). No significant differences were found between the two groups in VAS scores, CMSS-t scores, CMSS-s scores, SAS, or SDS scores for the three menstrual cycles at baseline (*p* > 0.05).

**TABLE 1:** Demographic and pain assessment of baseline and after treatment.

Items	Conditions	Verum acupuncture group (*n* = 20)	Sham acupuncture group (*n* = 14)	*T*	*p* *
Age (years)		24.70 ± 2.11	24.29 ± 1.90	0.59	0.56
Duration of PDM (years)		7.59 ± 2.91	7.84 ± 2.73	–0.26	0.80
Height (cm)		161.45 ± 4.06	160.71 ± 4.29	0.51	0.62
Weight (kg)		50.95 ± 3.81	49.75 ± 4.10	0.88	0.39
VAS scores	Pre-treatment	6.18 ± 0.99	5.93 ± 1.21	0.65	0.52
	Post-treatment	3.35 ± 1.50	5.39 ± 1.39	–	–
	Post-pre	–2.83 ± 1.57	–0.54 ± 1.55	–4.21	<0.001
CMSS-t scores	Pre-treatment	17.85 ± 8.36	18.50 ± 5.24	–0.26	0.80
	Post-treatment	13.20 ± 7.05	15.21 ± 5.28	–	–
	Post-pre	–4.65 ± 6.68	–3.29 ± 4.29	–0.67	0.51
CMSS-s scores	Pre-treatment	18.50 ± 7.74	14.43 ± 3.34	1.84	0.07
	Post-treatment	12.88 ± 7.19	14.50 ± 6.37	–	–
	Post-pre	–5.63 ± 7.80	0.07 ± 6.49	–2.24	0.03
SAS scores	Pre-treatment	41.88 ± 4.77	40.68 ± 8.08	0.54	0.59
	Post-treatment	35.44 ± 4.12	37.38 ± 5.48	–	–
	Post-pre	–6.44 ± 4.65	–3.30 ± 6.57	–1.63	0.11
SDS scores	Pre-treatment	40.36 ± 6.61	44.20 ± 9.97	–1.35	0.19
	Post-treatment	31.75 ± 5.34	39.91 ± 7.26	–	–
	Post-pre	–14.96 ± 4.27	–12.27 ± 7.30	–1.36	0.18

Values are mean ± standard deviation (SD); “post-pre” means changes in clinical outcomes before and after treatment; *p < 0.05 is considered statistically significant. PDM, primary dysmenorrhea; VAS, visual analog scale; SAS, self-anxiety scale; SDS, self-depression scale; CMSS-t, Cox Menstruation Symptom Scale-time subscale; CMSS-s, Cox Menstruation Symptom Scale-severity subscale.

#### Clinical outcomes

The VA group showed a significant decrease in VAS scores, CMSS-t scores, CMSS-s scores, SAS scores, and SDS scores after the treatment (*p* < 0.05). In contrast, paired *t*-sample tests revealed no significant changes in VAS scores, SAS scores, and CMSS-s scores (*p* < 0.05), a significant decrease in CMSS-t scores (*p* < 0.05) and an approach significant change in SDS (*p* = 0.05) after manipulating SA. RMANOVA suggested that the changes in VAS scores and CMSS-s scores were more significant in the VA group compared with the SA group; there were no significant differences in the changes in SAS, SDS, and CMSS-t scores between the two groups (see Table 1).

### Frequency-specific global functional connectivity density alternation

#### Post-pre-global functional connectivity density alternations

**FIGURE 1:**
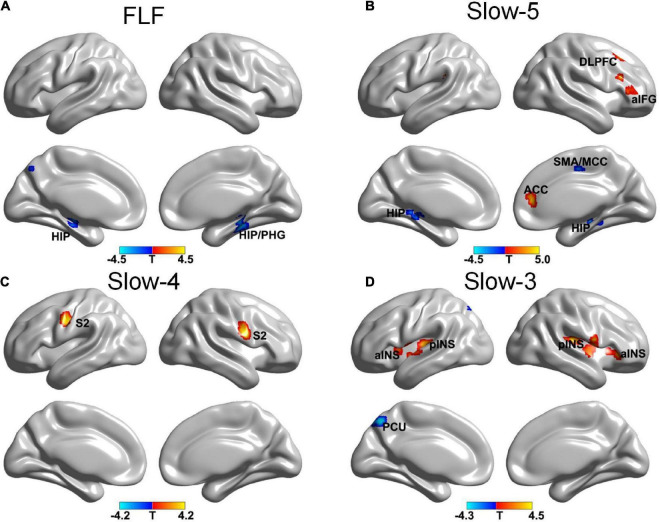
The gFCD distribution patterns at multiple frequency-bands following verum acupuncture (voxel-level p < 0.005, cluster-level p < 0.05, cluster size > 20 voxels, the small volume corrected and FWE corrected). **(A)** A decreased gFCD in HIP/PHG at FLF; **(B)** A decreased gFCD in HIP, MCC/SMA and an increased gFCD in ACC, DLPFC, and aIFG at Slow-5 band; **(C)** An increased gFCD in S2 at Slow-4 band. **(D)** A decreased gFCD in the PCU, and an increased gFCD in INS. gFCD, global functional connectivity density; HIP, hippocampus; PHG, parahippocampal gyrus; SMA, supplementary motor area; MCC, middle cingulate cortex; DLPFC, dorsolateral prefrontal cortex; S2, second somatosensory cortex; aINS, anterior INS; pINS, posterior INS; PCU, precuneus.

The more pronounced frequency-specific gFCD changes were observed in VA compared to SA after the treatment. The VA group showed significant gFCD decreases in the bilateral hippocampus at FLF. At Slow-5 band, the gFCD reduced in the hippocampus and right middle cingulate cortex (MCC)/supplementary motor area (SMA) bilaterally and increased in the right ACC, right DLPFC and right anterior inferior frontal gyrus (aIFG). An increased gFCD was seen in the S2 bilaterally within Slow-4 band. The Slow-3 band resulted in a decreased gFCD in the left precuneus (PCU) and an increased gFCD in the bilateral INS (see Figure 1 and Table 2).

**TABLE 2:** Frequency-specific gFCD alternations in the VA group.

Frequency band	Contrast	Cluster regions	L/R	Cluster size	MNI coordinates			*Z*-score
					***x***	***y***	***z***	
Full low frequency	Post > Pre	–						
	Pre > Post	HIP	L	20	0	–63	48	3.63
		HIP/PHG	R	36	27	–30	–18	3.44
Slow-5	Post > Pre	ACC	R	26	9	42	9	3.36
		DLPFC	R	20	33	24	45	3.73
		aIFG	R	32	51	39	9	3.63
	Pre > Post	HIP	L	43	–15	–33	–6	3.52
		HIP	R	30	36	–27	–15	3.22
		SMA/MCC	R	26	12	–12	48	3.54
Slow-4	Post > Pre	S2	L	59	–54	–6	39	3.46
		S2	R	58	63	3	18	3.30
	Pre > Post	–						
Slow-3	Post > Pre	aINS	L	37	–39	12	3	4.07
		aINS	R	22	39	30	–3	3.05
		pINS	L	148	–51	–6	3	4.09
		pINS	R	162	69	–3	9	3.76
	Pre > Post	PCU	L	34	–9	–66	51	3.52

Voxel level, p < 0.005, cluster level, p < 0.05, cluster size > 20 voxels; the small volume correction was applied in case the pain-related brain regions (cluster size less than or equal to 20) were not significant by FWE test. HIP, Hippocampus; PHG, Parahippocampal Gyrus; ACC, Anterior Cingulate Cortex; DLPFC, Dorsolateral Prefrontal Cortex; aIFG, Anterior Inferior Frontal Gyrus; SMA, Supplementary Motor Area; MCC, Middle Cingulate Cortex; S2, Second Somatosensory Cortex; aINS, Anterior Insula; pINS, Posterior insula; PCU, Precuneus.

**FIGURE 2:**
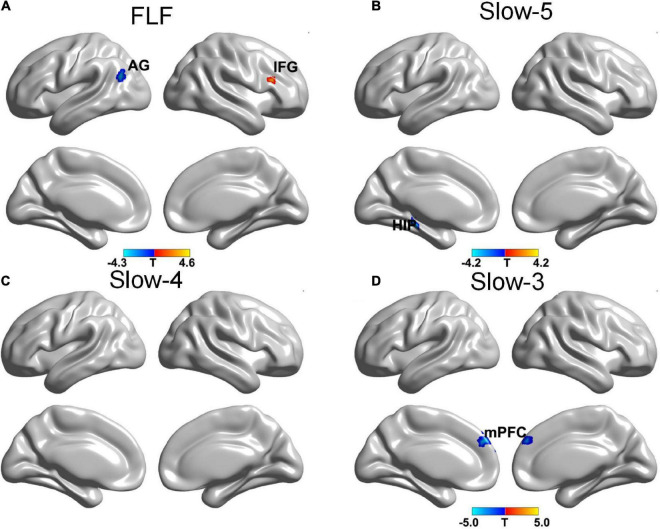
The gFCD distribution patterns at multiple frequency-bands following sham acupuncture (voxel-level p < 0.005, cluster-level p < 0.05, cluster size > 20 voxels, the small volume corrected and FWE corrected). **(A)** A decreased gFCD in AG, and an increased gFCD in IFG at FLF; **(B)** A decreased gFCD in HIP at Slow-5 band; **(C)** No significant change at Slow-4 band; **(D)** A decreased gFCD in mPFC at Slow-3 band. gFCD, global functional connectivity density; AG, angular gyrus; IFG, frontal inferior gyrus; HIP, hippocampus; mPFC, medial prefrontal cortex.

The SA group showed a decreased gFCD in the left angular gyrus (AG) and an increased gFCD in the right IFG at FLF. Also, a decreased gFCD was located in the left hippocampus at Slow-5 band. The gFCD in the bilateral medial prefrontal cortex (mPFC) reduced within the Slow-3 band (see Figure 2 and Table 3).

**TABLE 3:** Frequency-specific gFCD alternations in the SA group.

Frequency band	Contrast	Cluster regions	L/R	Cluster size	MNI coordinates			*Z*-score
					***x***	***y***	***z***	
Full low frequency	Post > Pre	IFG	R	26	54	27	24	3.66
	Pre > Post	AG	L	26	–51	–69	24	3.33
Slow-5	Post > Pre	–						
	Pre > Post	HIP	L	34	–24	–30	–18	3.27
Slow-4	Post > Pre	–						
	Pre > Post	–						
Slow-3	Post > Pre	–						
	Pre > Post	mPFC	L/R	95	–3	51	39	3.66

Voxel level, p < 0.005, cluster level, p < 0.05, cluster size > 20 voxels; the small volume correction was applied in case the pain-related brain regions (cluster size less than or equal to 20) were not significant by FWE test. Abbreviations: IFG, Inferior Frontal Gyrus; AG, Angular Gyrus; HIP, Hippocampus; mPFC, Medial Prefrontal Cortex.

#### Comparison of global functional connectivity density within groups

The RMANOVA explored the more significant-altered brain regions at different frequency bands in VA compared with SA. The VA increased more gFCD in the left DLPFC at Slow-5 band and in the left MCC at Slow-4 band (Supplementary Figure 3 and Supplementary Table 1). Compared with SA, VA decreased more gFCD in the left caudate nucleus (CAU), nucleus accumbens (NAC), the right hippocampus and hippocampal gyrus (PHG) by VA at FLF; in the left CAU, NAC, and the aIFG was significant at Slow-4 band; on the left hippocampus and right SMA at Slow-3 band (Supplementary Figure 3 and Supplementary Table 1).

#### Correlation analysis results

**FIGURE 3:**
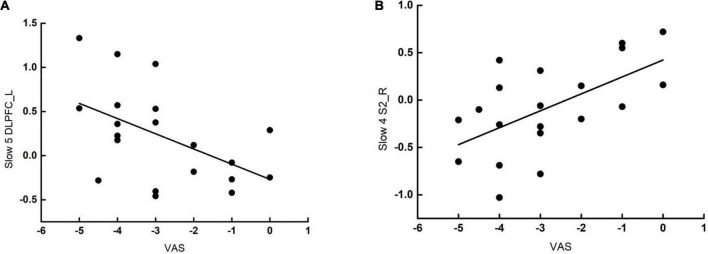
Correlation analysis following verum acupuncture and VAS scores of PDM. **(A)** The gFCD changes at Slow-5 band of the left DLPFC in patients with PDM was negatively correlated with the change in VAS scores (r = –0.508, p = 0.022). **(B)** The gFCD changes at Slow-4 band of the right S2 in patients with PDM was positively correlated with the change in VAS scores (r = –0.587, p = 0.006). gFCD, global functional connectivity density; VAS, visual analog scale; S2, second somatosensory cortex; DLPFC, dorsolateral prefrontal cortex; pINS, posterior INS; aINS, anterior INS; ACC, anterior cingulate gyrus.

We investigated the clinical significance of gFCD alterations with acupuncture in the VA group only. The change in gFCD values at Slow-5 band of the left DLPFC in patients with PDM treated by VA was negatively correlated with the change in VAS scores (*r* = –0.508, *p* = 0.022). Moreover, the gFCD changes at Slow-4 band of the S2 was positively correlated with the change in VAS scores (*r* = 0.587, *p* = 0.006) (see Figure 3).

## Discussion

This study investigated the frequency-dependent gFCD alternations before and after VA and SA treatment. Generally, the Slow-5 band and Slow-4 band indicated gray-matter-related brain function changes (Zuo et al., 2010), whereas a spontaneous brain activity at the Slow-3 band can predict the lower frequency node activity (Bajaj et al., 2013). The VA group demonstrated a substantial reduction in pain intensity and improvement in PDM symptoms when compared to the SA group. Remarkably, the VA group raised gFCD in the DLPFC at the Slow-5 band and decreased gFCD in the hippocampus at the Slow-3 band, which was in contrast to the direction of pathological gFCD brain function changes in the DLPFC and hippocampus observed in our earlier study (Yu et al., 2021). The results also indicated that VA modulated a more comprehensive range of brain regions than SA. The correlation analysis showed gFCD change in DLPFC at Slow-5 band was negatively correlated with the change of VAS scores in patients with PDM following VA. As a result, the DLPFC and hippocampus may be therapeutic targets for re-establishing normal brain activity in patients with PDM.

The result showed an altered gFCD in the VA group located in S2, CEN (including the DLPFC, ACC, MCC, and aIFG), DMN (including PCU), INS, hippocampus, and PHG. The result is consistent with the previously reported brain responses to VA, down-regulating hippocampus, SMA, and PCU and up-regulating S2, INS, ACC, and DLPFC were observed (Ming-Ting et al., 1999; Bai and Lao, 2013). As a result, brain adaption to VA overlapped with several core networks, involving sensory and cognitive function in pain processing progress, which indicated the non-specific modulation mechanism of acupuncture.

The hippocampus and DLPFC were identified as frequency-specific therapeutic targets for specific acupuncture-based PDM modulation. Our prior study showed a decreased gFCD in DLPFC at Slow-5 band and an increased gFCD in hippocampus at Slow-3 band in patients with PDM when compared with healthy controls (Yu et al., 2021). In contrast, the current findings indicated ascending DLPFC at Slow-5 band and descending hippocampus at Slow-3 band following VA. Both DLPFC and hippocampus are crucial nodes of pain processing. The DLPFC regulates top–down pain pathways in the human brain, which is intimately connected to the individual’s executive function (Eippert et al., 2009; Kong et al., 2013). As a result, DLPFC is capable of exerting cognitive control over the perception of pain. Fierro et al. (2010) performed a non-invasive short-duration high-frequency repetitive transcranial magnetic stimulation (rTMS) of the left DLPFC considerably diminish the pain perception generated by capsaicin stimulation. Additionally, studies have revealed a link between pain self-control and DLPFC downregulation in patients with PDM (Lorenz et al., 2003). Therefore, the overlap of DLPFC in this study implied that acupuncture could alleviate pain by modulating cognition to reach individual pain control in PDM. The deficits in hippocampus volume, neurogenesis, and synaptic plasticity are connected with abnormal emotional functioning associated with various types of chronic pain (Mutso et al., 2012; Grilli, 2017). These findings suggested that the hippocampus may regulate pain by modulating pain perception and emotional memory (Rolls, 2015). The PDM has been exacerbated by increased emotional stress caused by aberrant feedback from the hippocampus to the hypothalamus (Jacobson and Sapolsky, 1991; Wang et al., 2004; Ulrich-Lai et al., 2006).

Consistent with the results of the previous studies, we found that acupuncture could down-regulate the hippocampus (Gao et al., 2021; Pang et al., 2021). We speculated that acupuncture could help diminish hippocampal sensitivity and reduce unpleasant memories of pain in PDM (Egorova et al., 2015).

Moreover, S2 is involved in the regulation of nociception and the encoding of painful experiences (Schreckenberger et al., 2005). Consistent with prior research, VA activated S2 (Maeda et al., 2013). Additionally, VA was consistently demonstrated resting-state down-regulation of the DMN subregion (Jung et al., 2015; Makary et al., 2018). Intriguingly, we discovered that PCU was deactivated following VA in our investigation. The PCU is recognized as the hub of the DMN, modulating a broad range of highly interconnected functions and regulating pain, affection, and empathy (Cavanna and Trimble, 2006). The consistent down-regulation in DMN was also shown in the study of Zou et al. (2019) that FC within the DMN (between left superior prefrontal cortex and left PCU) after acupuncture decreased to HC levels. The INS plays an essential role in pain intensity coding. Studies have revealed a decreased gray matter density in the INS of patients with PDM (Tan et al., 2017). Spontaneous brain activity at the Slow-3 band is associated with structural correlation (Bajaj et al., 2013). Our study up-regulated INS in Slow-3, suggesting that VA may modulate INS structure. Although the current research has confirmed the modulation of INS function by acupuncture, the modulation of INS structure by acupuncture requires more investigation (Jung et al., 2015; Dun et al., 2017; Guo et al., 2019; Duan et al., 2021). In addition, the modulation of the cingulate cortex (ACC, MCC) and IFG by acupuncture are associated with modulation of pain perception (Fuchs et al., 2014) and empathic processing of pain (Li et al., 2021).

The results showed that VA and SA had distinct effects on the brain responses of patients with PDM. The prior studies have identified the distinctions and consistency in the brain response to VA compared to SA (Fang et al., 2009; Harris et al., 2009; Chae et al., 2013; Maeda et al., 2017). As with VA on brain modulation, SA down-regulates gFCD in the left hippocampus and up-regulates gFCD in the right IFG. The result also showed that SA reduced gFCD in the anterior DMN. The result implied that SA, similar to VA, could modulate pain emotions in patients with PDM. The studies on SA or phantom acupuncture have concluded that SA can specifically activate DLPFC to provide the placebo effect (Makary et al., 2018; Shi et al., 2021). However, this study suggested that SA did not exhibit a placebo effect associated with self-control in pain. The medial prefrontal lobe is primarily involved in the modulation of visceral motor output of the individual’s internal experience and is associated with pain rumination (Kucyi et al., 2014). The SA modulation of the medial prefrontal cortex suggests that the placebo effect of SA may be mediated by improving individual pain rumination.

We preliminarily investigated the modulation of acupuncture on PDM at different frequency bands. The gFCD provided a mapping of the functional connectivity between the brain regions throughout the brain and is more sensitive to identifying the individual differences (Tomasi and Volkow, 2010, 2011). The result showed that VA modulated more comprehensive frequency than SA. At the Slow-5 band, VA on the left DLPFC increased significantly compared to SA. Similar to a previous longitudinal study, reduced amplitude of low-frequency fluctuation (ALFF)/fALFF in DLPFC patients with trigeminal neuralgia was observed exclusively at the Slow-5 band (Zhang et al., 2019). At FLF and Slow-3 band, decreased gFCD was more significant in the hippocampus in VA rather than SA. Above results suggested that modulation in different frequency bands is more significant in VA than in SA, and there is a frequency-specific sensitivity of the same modulated brain regions. Notably, abnormal BOLD signal fluctuations occur in different frequency bands ranging from 0.1 to 0.25 Hz under different chronic pain conditions (Baliki et al., 2011b; Alshelh et al., 2016). However, the neurophysiological mechanisms behind the different frequency bands are largely unknown, which needs further exploration (Buzsaki and Draguhn, 2004).

This study found that the gFCD changes of left DLPFC at Slow-5 band were negatively correlated with the change in pain VAS scores, suggesting that VA could increase the cognitive modulation of DLPFC to improve the control of pain in individuals (Lorenz et al., 2003). Our study found that more gFCD changes in DLPFC correspond to more reduction in VAS scores. This suggests that the changes in gFCD of DLPFC are consistent with improvements in VAS and that VA may increase individual control in pain intensity by improving the cognitive modulation of DLPFC. The increased gFCD of S2 after VA also associated with the pain relief of PDM. Therefore, the response of DLPFC and S2 after VA may be an objective biomarker that can predict the efficacy of acupuncture in the treatment of PDM.

## Limitations

There are still limitations to the study. First, the study design was small sample size, and patient compliance was influenced by the schedule of the MRI scan (3 days before the onset of menstruation). Second, this study focused exclusively on the altered FCD of the PDM brain network, and more substantial structural and metabolic brain changes need to be investigated. Third, the results showed that acupuncture normalized the brain activity of DLPFC in PDM. However, no relevant behavioral scales in this study were designed to assess individual executive function in this study.

## Conclusion

In summary, we re-verified that VA is significantly more effective than SA in treating PDM. The findings supported the hypothesis that acupuncture can restore normalcy to the DLPFC and hippocampus, which had previously been identified as abnormal in PDM. The results elucidated the brain targets of acupuncture for PDM and may facilitate the development of brain stimulation methods to facilitate the therapeutic response to acupuncture.

## Data availability statement

All data are presented in this article, and the raw data are not available to the public. Requests to access the datasets should be directed to JY, jenny_yang_jie@126.com.

## Ethics statement

The study protocol was approved by the Medical Ethics Review of the Sichuan Regional Ethics Review Committee of Traditional Chinese Medicine (No. 2013KL-033). The patients/participants provided their written informed consent to participate in this study.

## Author contributions

JY and S-YY: experimental design. L-YL, XL, Z-LT, and QZ: data collection. S-YY and Z-FS: data analysis. L-YL, S-YY, and Z-LT: manuscript preparation and revision. All authors contributed to the article and approved the submitted version.

## Footnotes

1
http://www.fil.ion.ucl.ac.uk/spm

2
http://www.brainnetome.org/toolkit/bf/

3
https://afni.nimh.nih.gov/pub/dist/doc/program_help/3dClustSim.html

## Abbreviations

AG: angular gyrus
ACC: anterior cingulate cortex
aIFG: anterior inferior frontal gyrus
CAU: caudate nucleus
CEN: central executive network
CMSS: Cox Menstrual Symptom Scale
CMSS-s: Cox Menstrual Symptom Scale-severity
CMSS-t: Cox Menstrual Symptom Scale-time
DMN: default mode network
DLPFC: dorsolateral prefrontal cortex
TE: echo time
FOV: field of view
FLF: full low frequency
FCD: functional connectivity density
gFCD: global functional connectivity density
PHG: hippocampal gyrus
mPFC: medial prefrontal cortex
MCC: middle cingulate cortex
MNI: Montreal Neurological Institute
NAC: nucleus accumbens
PCU: precuneus
PDM: primary dysmenorrhea
S1: primary somatosensory cortex
RMANOVA: repeated measurement of analysis of variance
TR: repetition time
rTMS: repetitive transcranial magnetic stimulation
rs-fMRI: resting-state functional magnetic resonance imaging
S2: second somatosensory cortex
SMN: sensorimotor network
SA: sham acupuncture
SMA: supplementary motor area
SAS: the Zung Self-Rating Anxiety Scale
SDS: the Zung Self-Rating Depression Scale
VA: verum acupuncture
VAS: visual analog scale.

## References

[B1] Abaraogu U. O. Igwe S. E. Tabansi-Ochiogu C. S. (2016). Effectiveness of SP6 (Sanyinjiao) acupressure for relief of primary dysmenorrhea symptoms: A systematic review with meta- and sensitivity analyses. *Complement. Ther. Clin. Pract.* 25 92–105. 10.1016/j.ctcp.2016.09.003 27863617

[B2] Alshelh Z. Di Pietro F. Youssef A. M. Reeves J. M. Macey P. M. Vickers E. R. (2016). Chronic Neuropathic Pain: It’s about the Rhythm. *J. Neurosci.* 36 1008–1018. 10.1523/JNEUROSCI.2768-15.2016 26791228 PMC6602000

[B3] Bai L. Lao L. (2013). Neurobiological foundations of acupuncture: the relevance and future prospect based on neuroimaging evidence. *Evid. Based Complement. Alternat. Med.* 2013:812568. 10.1155/2013/812568 23737848 PMC3666300

[B4] Bajaj S. Adhikari B. M. Dhamala M. (2013). Higher frequency network activity flow predicts lower frequency node activity in intrinsic low-frequency BOLD fluctuations. *PLoS One* 8:e64466. 10.1371/journal.pone.0064466 23691225 PMC3655147

[B5] Baliki M. N. Baria A. T. Apkarian A. V. (2011a). The cortical rhythms of chronic back pain. *J. Neurosci.* 31 13981–13990. 10.1523/JNEUROSCI.1984-11.2011 21957259 PMC3214084

[B6] Baliki M. N. Schnitzer T. J. Bauer W. R. Apkarian A. V. (2011b). Brain morphological signatures for chronic pain. *PLoS One* 6:e26010. 10.1371/journal.pone.0026010 22022493 PMC3192794

[B7] Berman B. M. Lao L. Langenberg P. Lee W. L. Gilpin A. M. Hochberg M. C. (2004). Effectiveness of acupuncture as adjunctive therapy in osteoarthritis of the knee: a randomized, controlled trial. *Ann. Intern. Med.* 141 901–910. 10.7326/0003-4819-141-12-200412210-00006 15611487

[B8] Buzsaki G. Draguhn A. (2004). Neuronal oscillations in cortical networks. *Science* 304 1926–1929. 10.1126/science.1099745 15218136

[B9] Cai R. L. Shen G. M. Wang H. Guan Y. Y. (2018). Brain functional connectivity network studies of acupuncture: a systematic review on resting-state fMRI. *J. Integr. Med.* 16 26–33. 10.1016/j.joim.2017.12.002 29397089

[B10] Cavanna A. E. Trimble M. R. (2006). The precuneus: a review of its functional anatomy and behavioural correlates. *Brain* 129 564–583. 10.1093/brain/awl004 16399806

[B11] Chae Y. Chang D. S. Lee S. H. Jung W. M. Lee I. S. Jackson S. (2013). Inserting needles into the body: a meta-analysis of brain activity associated with acupuncture needle stimulation. *J. Pain* 14 215–222. 10.1016/j.jpain.2012.11.011 23395475

[B12] Ciric R. Wolf D. H. Power J. D. Roalf D. R. Baum G. L. Ruparel K. (2017). Benchmarking of participant-level confound regression strategies for the control of motion artifact in studies of functional connectivity. *Neuroimage* 154 174–187. 10.1016/j.neuroimage.2017.03.020 28302591 PMC5483393

[B13] Cox D. J. Meyer R. G. (1978). Behavioral treatment parameters with primary dysmenorrhea. *J. Behav. Med.* 1 297–310. 10.1007/BF00846681 40034

[B14] Duan G. Chen Y. Pang Y. Feng Z. Liao H. Liu H. (2021). Altered fractional amplitude of low-frequency fluctuation in women with premenstrual syndrome via acupuncture at Sanyinjiao (SP6). *Ann. Gen. Psychiat.* 20:29. 10.1186/s12991-021-00349-z 33964936 PMC8106846

[B15] Dun W. H. Yang J. Yang L. Ding D. Ma X. Y. Liang F. L. (2017). Abnormal structure and functional connectivity of the anterior insula at pain-free periovulation is associated with perceived pain during menstruation. *Brain Imag. Behav.* 11 1787–1795. 10.1007/s11682-016-9646-y 27832449

[B16] Egorova N. Gollub R. L. Kong J. (2015). Repeated verum but not placebo acupuncture normalizes connectivity in brain regions dysregulated in chronic pain. *Neuroimag. Clin.* 9 430–435. 10.1016/j.nicl.2015.09.012 26594625 PMC4596925

[B17] Eippert F. Bingel U. Schoell E. D. Yacubian J. Klinger R. Lorenz J. (2009). Activation of the opioidergic descending pain control system underlies placebo analgesia. *Neuron* 63 533–543. 10.1016/j.neuron.2009.07.014 19709634

[B18] Fang J. Jin Z. Wang Y. Li K. Kong J. Nixon E. E. (2009). The salient characteristics of the central effects of acupuncture needling: limbic-paralimbic-neocortical network modulation. *Hum. Brain Mapp.* 30 1196–1206. 10.1002/hbm.20583 18571795 PMC6871074

[B19] Fierro B. De Tommaso M. Giglia F. Giglia G. Palermo A. Brighina F. (2010). Repetitive transcranial magnetic stimulation (rTMS) of the dorsolateral prefrontal cortex (DLPFC) during capsaicin-induced pain: modulatory effects on motor cortex excitability. *Exp. Brain Res.* 203 31–38. 10.1007/s00221-010-2206-6 20232062

[B20] Fomberstein K. Qadri S. Ramani R. (2013). Functional MRI and pain. *Curr. Opin. Anaesthesiol.* 26 588–593. 10.1097/01.aco.0000433060.59939.fe 23995063

[B21] Fox M. D. Raichle M. E. (2007). Spontaneous fluctuations in brain activity observed with functional magnetic resonance imaging. *Nat. Rev. Neurosci.* 8 700–711. 10.1038/nrn2201 17704812

[B22] Fuchs P. N. Peng Y. B. Boyette-Davis J. A. Uhelski M. L. (2014). The anterior cingulate cortex and pain processing. *Front. Integr. Neurosci.* 8:35. 10.3389/fnint.2014.00035 24829554 PMC4017137

[B23] Gao L. Zhang J. F. Williams J. P. Yan Y. N. Xiao X. L. Shi W. R. (2021). Neuropathic Pain Creates Systemic Ultrastructural Changes in the Nervous System Corrected by Electroacupuncture but Not by Pregabalin. *J. Pain Res.* 14 2893–2905. 10.2147/JPR.S322964 34548816 PMC8449649

[B24] Grilli M. (2017). Chronic pain and adult hippocampal neurogenesis: translational implications from preclinical studies. *J. Pain Res.* 10 2281–2286. 10.2147/JPR.S146399 29033604 PMC5614764

[B25] Guo J. Yang M. Biswal B. B. Yang P. Liao W. Chen H. (2019). Abnormal functional connectivity density in post-stroke aphasia. *Brain Topogr.* 32 271–282. 10.1007/s10548-018-0681-4 30293180

[B26] Harris R. E. Zubieta J. K. Scott D. J. Napadow V. Gracely R. H. Clauw D. J. (2009). Traditional Chinese acupuncture and placebo (sham) acupuncture are differentiated by their effects on mu-opioid receptors (MORs). *Neuroimage* 47 1077–1085. 10.1016/j.neuroimage.2009.05.083 19501658 PMC2757074

[B27] Hershman D. L. Unger J. M. Greenlee H. Capodice J. L. Lew D. L. Darke A. K. (2018). Effect of acupuncture vs sham acupuncture or waitlist control on joint pain related to aromatase inhibitors among women with early-stage breast cancer: A randomized clinical trial. *Jama* 320 167–176. 10.1001/jama.2018.8907 29998338 PMC6583520

[B28] Hinman R. S. McCrory P. Pirotta M. Relf I. Forbes A. Crossley K. M. (2014). Acupuncture for chronic knee pain: A randomized clinical trial. *Jama* 312 1313–1322. 10.1001/jama.2014.12660 25268438

[B29] Jacobson L. Sapolsky R. (1991). The role of the hippocampus in feedback regulation of the hypothalamic-pituitary-adrenocortical axis. *Endocr. Rev.* 12 118–134. 10.1210/edrv-12-2-118 2070776

[B30] Jung W. M. I Lee S. Wallraven C. Ryu Y. H. Park H. J. Chae Y. (2015). Cortical activation patterns of bodily attention triggered by acupuncture stimulation. *Sci. Rep.* 5:12455. 10.1038/srep12455 26211895 PMC4515634

[B31] Kilpatrick L. A. Kutch J. J. Tillisch K. Naliboff B. D. Labus J. S. Jiang Z. (2014). Alterations in resting state oscillations and connectivity in sensory and motor networks in women with interstitial cystitis/painful bladder syndrome. *J. Urol.* 192 947–955. 10.1016/j.juro.2014.03.093 24681331 PMC4432915

[B32] Kong J. Gollub R. Huang T. Polich G. Napadow V. Hui K. (2007). Acupuncture de qi, from qualitative history to quantitative measurement. *J. Altern. Complement. Med.* 13 1059–1070. 10.1089/acm.2007.0524 18166116

[B33] Kong J. Jensen K. Loiotile R. Cheetham A. Wey H. Y. Tan Y. (2013). Functional connectivity of the frontoparietal network predicts cognitive modulation of pain. *Pain* 154 459–467. 10.1016/j.pain.2012.12.004 23352757 PMC3725961

[B34] Kucyi A. Moayedi M. Weissman-Fogel I. Goldberg M. B. Freeman B. V. Tenenbaum H. C. (2014). Enhanced medial prefrontal-default mode network functional connectivity in chronic pain and its association with pain rumination. *J. Neurosci.* 34 3969–3975. 10.1523/JNEUROSCI.5055-13.2014 24623774 PMC6705280

[B35] Lefebvre G. Pinsonneault O. Antao V. Black A. Burnett M. Feldman K. (2005). Primary dysmenorrhea consensus guideline. *J. Obstet. Gynaecol. Can.* 27 1117–1146. 10.1016/s1701-2163(16)30395-4 16524531

[B36] Li Y. Li W. Zhang T. Zhang J. Jin Z. Li L. (2021). Probing the role of the right inferior frontal gyrus during Pain-Related empathy processing: Evidence from fMRI and TMS. *Hum. Brain Mapp.* 42 1518–1531. 10.1002/hbm.25310 33283946 PMC7927301

[B37] Liu M. G. Chen J. (2009). Roles of the hippocampal formation in pain information processing. *Neurosci. Bull.* 25 237–266. 10.1007/s12264-009-0905-4 19784080 PMC5552607

[B38] Lorenz J. Minoshima S. Casey K. L. (2003). Keeping pain out of mind: the role of the dorsolateral prefrontal cortex in pain modulation. *Brain* 126 1079–1091. 10.1093/brain/awg102 12690048

[B39] Maeda Y. Kettner N. Lee J. Kim J. Cina S. Malatesta C. (2013). Acupuncture evoked response in contralateral somatosensory cortex reflects peripheral nerve pathology of carpal tunnel syndrome. *Med. Acupunct.* 25 275–284. 10.1089/acu.2013.0964 24761177 PMC3746237

[B40] Maeda Y. Kim H. Kettner N. Kim J. Cina S. Malatesta C. (2017). Rewiring the primary somatosensory cortex in carpal tunnel syndrome with acupuncture. *Brain* 140 914–927. 10.1093/brain/awx015 28334999 PMC5837382

[B41] Makary M. M. Lee J. Lee E. Eun S. Kim J. Jahng G. H. (2018). Phantom acupuncture induces placebo credibility and vicarious sensations: A parallel fmri study of low back pain patients. *Sci. Rep.* 8:930. 10.1038/s41598-017-18870-1 29343693 PMC5772373

[B42] McCormack H. M. Horne D. J. Sheather S. (1988). Clinical applications of visual analogue scales: a critical review. *Psychol. Med.* 18 1007–1019. 10.1017/s0033291700009934 3078045

[B43] Ming-Ting W. Hsieh J.-C. Xiong J. (1999). Central nervous pathway for acupuncture stimulation: Localization of processing with functional MR imaging of the brain—preliminary experience. *Radiology* 212 133–141. 10.1148/radiology.212.1.r99jl04133 10405732

[B44] Mutso A. A. Radzicki D. Baliki M. N. Huang L. Banisadr G. Centeno M. V. (2012). Abnormalities in hippocampal functioning with persistent pain. *J. Neurosci.* 32 5747–5756. 10.1523/JNEUROSCI.0587-12.2012 22539837 PMC3365570

[B45] Pang Y. Liao H. Duan G. Feng Z. Liu H. Zou Z. (2021). Regulated aberrant amygdala functional connectivity in premenstrual syndrome via electro-acupuncture stimulation at sanyinjiao acupoint(SP6). *Gynecol. Endocrinol.* 37 315–319. 10.1080/09513590.2020.1855633 33307896

[B46] Rogachov A. Cheng J. C. Hemington K. S. Bosma R. L. Kim J. A. Osborne N. R. (2018). Abnormal low-frequency oscillations reflect trait-like pain ratings in chronic pain patients revealed through a machine learning approach. *J. Neurosci.* 38 7293–7302. 10.1523/JNEUROSCI.0578-18.2018 30012686 PMC6596036

[B47] Rolls E. T. (2015). Limbic systems for emotion and for memory, but no single limbic system. *Cortex* 62 119–157. 10.1016/j.cortex.2013.12.005 24439664

[B48] Schmidt-Wilcke T. (2015). Neuroimaging of chronic pain. *Best Pract. Res. Clin. Rheumatol.* 29 29–41. 10.1016/j.berh.2015.04.030 26266997

[B49] Schmidt-Wilcke T. Diers M. (2017). New insights into the pathophysiology and treatment of fibromyalgia. *Biomedicines* 5:22. 10.3390/biomedicines5020022 28536365 PMC5489808

[B50] Schreckenberger M. Siessmeier T. Viertmann A. Landvogt C. Buchholz H. G. Rolke R. (2005). The unpleasantness of tonic pain is encoded by the insular cortex. *Neurology* 64 1175–1183. 10.1212/01.WNL.0000156353.17305.52 15824343

[B51] Shi Y. Cui S. Zeng Y. Huang S. Cai G. Yang J. (2021). Brain network to placebo and nocebo responses in acute experimental lower back pain: A multivariate granger causality analysis of fMRI data. *Front. Behav. Neurosci.* 15:696577. 10.3389/fnbeh.2021.696577 34566591 PMC8458622

[B52] Tan L. L. Pelzer P. Heinl C. Tang W. Gangadharan V. Flor H. (2017). A pathway from midcingulate cortex to posterior insula gates nociceptive hypersensitivity. *Nat. Neurosci.* 20 1591–1601. 10.1038/nn.4645 28920932

[B53] Tomasi D. Shokri-Kojori E. Volkow N. D. (2016). Temporal changes in local functional connectivity density reflect the temporal variability of the amplitude of low frequency fluctuations in gray matter. *PLoS One* 11:e0154407. 10.1371/journal.pone.0154407 27116610 PMC4846007

[B54] Tomasi D. Volkow N. D. (2010). Functional connectivity density mapping. *Proc. Natl. Acad. Sci. U. S. A.* 107 9885–9890. 10.1073/pnas.1001414107 20457896 PMC2906909

[B55] Tomasi D. Volkow N. D. (2011). Functional connectivity hubs in the human brain. *Neuroimage* 57 908–917. 10.1016/j.neuroimage.2011.05.024 21609769 PMC3129362

[B56] Tomasi D. Volkow N. D. (2014). Mapping small-world properties through development in the human brain: disruption in schizophrenia. *PLoS One* 9:e96176. 10.1371/journal.pone.0096176 24788815 PMC4005771

[B57] Tu Y. Jung M. Gollub R. L. Napadow V. Gerber J. Ortiz A. (2019). Abnormal medial prefrontal cortex functional connectivity and its association with clinical symptoms in chronic low back pain. *Pain* 160 1308–1318. 10.1097/j.pain.0000000000001507 31107712 PMC6530583

[B58] Ulrich-Lai Y. M. Xie W. Meij J. T. Dolgas C. M. Yu L. Herman J. P. (2006). Limbic and HPA axis function in an animal model of chronic neuropathic pain. *Physiol. Behav.* 88 67–76. 10.1016/j.physbeh.2006.03.012 16647726

[B59] Wang L. Wang X. Wang W. Chen C. Ronnennberg A. G. Guang W. (2004). Stress and dysmenorrhoea: a population based prospective study. *Occup. Environ. Med.* 61 1021–1026. 10.1136/oem.2003.012302 15550609 PMC1740691

[B60] Wang Y. Xu J. Zhang Q. Zhang Q. Yang Y. Wei W. (2021). Immediate analgesic effect of acupuncture in patients with primary dysmenorrhea: A fMRI study. *Front. Neurosci.* 15:647667. 10.3389/fnins.2021.647667 34108856 PMC8180846

[B61] Witt C. M. Reinhold T. Brinkhaus B. Roll S. Jena S. Willich S. N. (2008). Acupuncture in patients with dysmenorrhea: a randomized study on clinical effectiveness and cost-effectiveness in usual care. *Am. J. Obstet. Gynecol.* 198 166.e1–8. 10.1016/j.ajog.2007.07.041 18226614

[B62] Worsley K. J. Marrett S. Neelin P. Vandal A. C. Friston K. J. Evans A. C. (1996). A unified statistical approach for determining significant signals in images of cerebral activation. *Hum. Brain Mapp.* 4 58–73. 10.1002/(SICI)1097-019319964:1<58::AID-HBM4>3.0.CO;2-O 20408186

[B63] Yu S. Xu J. Shen Z. Wang Y. Wei W. Guo X. (2021). Frequency-specific alterations in brain function in patients with primary dysmenorrhea. *Pain Med.* 23 902–911. 10.1093/pm/pnab225 34314503

[B64] Yu S. Y. Lv Z. T. Zhang Q. Yang S. Wu X. Hu Y. P. (2017). Electroacupuncture is beneficial for primary dysmenorrhea: The evidence from meta-analysis of randomized controlled trials. *Evid. Based Complement. Alternat. Med.* 2017:1791258. 10.1155/2017/1791258 29358960 PMC5735637

[B65] Zhang J. Zhang Y. Hu L. Huang X. Liu Y. Li J. (2020). Global trends and performances of magnetic resonance imaging studies on acupuncture: A bibliometric analysis. *Front. Neurosci.* 14:620555. 10.3389/fnins.2020.620555 33551731 PMC7854454

[B66] Zhang Y. Mao Z. Pan L. Ling Z. Liu X. Zhang J. (2019). Frequency-specific alterations in cortical rhythms and functional connectivity in trigeminal neuralgia. *Brain Imaging Behav.* 13 1497–1509. 10.1007/s11682-019-00105-8 31209834

[B67] Zou Y. Tang W. Li X. Xu M. Li J. (2019). Acupuncture reversible effects on altered default mode network of chronic migraine accompanied with clinical symptom relief. *Neural. Plast.* 2019:5047463. 10.1155/2019/5047463 31011330 PMC6442315

[B68] Zung W. W. (1971). A rating instrument for anxiety disorders. *Psychosomatics* 12 371–379. 10.1016/S0033-3182(71)71479-0 5172928

[B69] Zung W. W. Richards C. B. Short M. J. (1965). Self-rating depression scale in an outpatient clinic. Further validation of the SDS. *Arch. Gen. Psychiat.* 13 508–515. 10.1001/archpsyc.1965.01730060026004 4378854

[B70] Zuo X. N. Di Martino A. Kelly C. Shehzad Z. E. Gee D. G. Klein D. F. (2010). The oscillating brain: complex and reliable. *Neuroimage* 49 1432–1445. 10.1016/j.neuroimage.2009.09.037 19782143 PMC2856476

