# Supplementary material for: Acupuncture modulates the frequency-specific functional connectivity density in primary dysmenorrhea
Source: Front Neurosci. 2022 Aug 16;16:917721. doi: 10.3389/fnins.2022.917721 (PMC9426343; doi:10.3389/fnins.2022.917721)
Supplement: Supplementary file 1 [file Data_Sheet_1.PDF]

Suplementary table

Table S1. Frequency-specific gFCD changes comparison between the VA group and the SA group.

Frequency band	Contrast	Cluster regions	L/R	Cluster size	MNI coordinates			Z score
					x	y	z	
Brain regions with increased gFCD post-treatment								
Full low frequency	VA>SA	-						
Slow5	VA>SA	DLPFC	L	41	-39	30	24	3.37
Slow4	VA>SA	MCC	L	64	-12	-21	36	4.09
Slow3	VA>SA	-						
Brain regions with decreased gFCD post-treatment								
Full low frequency	VA>SA	CAU/NAC	L	88	-12	6	-3	3.72
		HIP/PHG	R	58	33	-33	-15	3.40
Slow5	VA>SA	-						
Slow4	VA>SA	aIFG	R	28	54	42	9	3.48
		CAU/NAC	L	58	-12	9	-6	3.36
Slow3	VA>SA	SMA	R	22	9	-15	51	2.93
		HIP	L	31	-27	-6	-12	3.52

Note: Voxel level, $p < 0.005$, cluster level, $p < 0.05$, cluster size >20 voxels; the small volume correction was applied in case the pain-related brain regions (cluster size less than or equal to 20) were not significant by FWE test. Abbreviations: VA. verum acupuncture; SA, sham acupuncture; DLPFC, Dorsolateral Prefrontal Cortex; MCC, Middle Cingulate Cortex; CAU, Caudate Nucleus; NAC, Nucleus Accumbens; HIP, Hippocampus; PHG, Parahippocampal Gyrus; aIFG, Anterior Inferior Frontal Gyrus; SMA, Supplementary Motor Area.

Suplementary figure

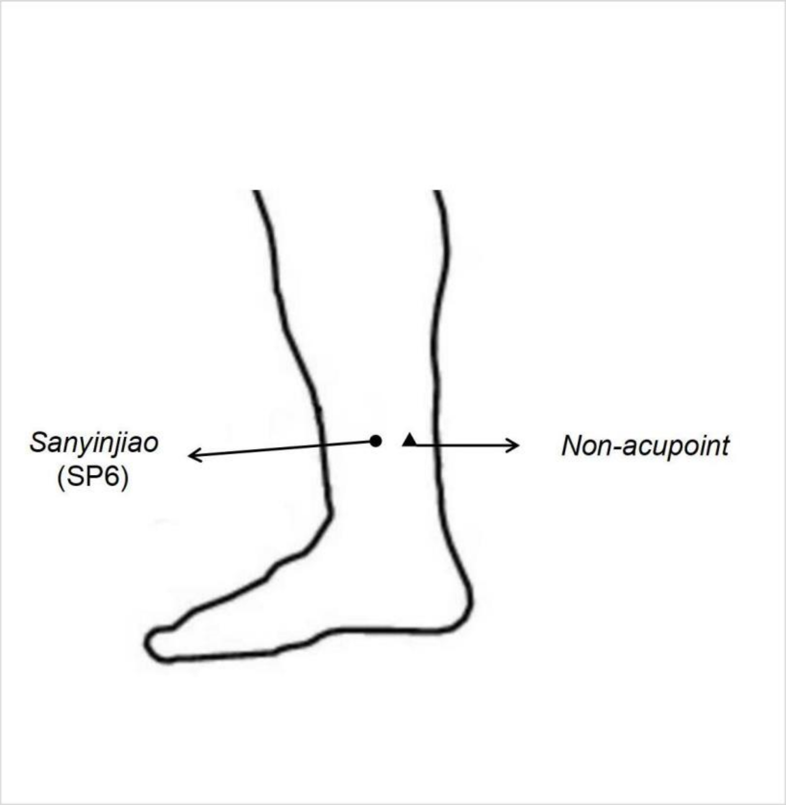

Figure S1. Locations of acupoints applied in the verum and sham acupuncture groups.

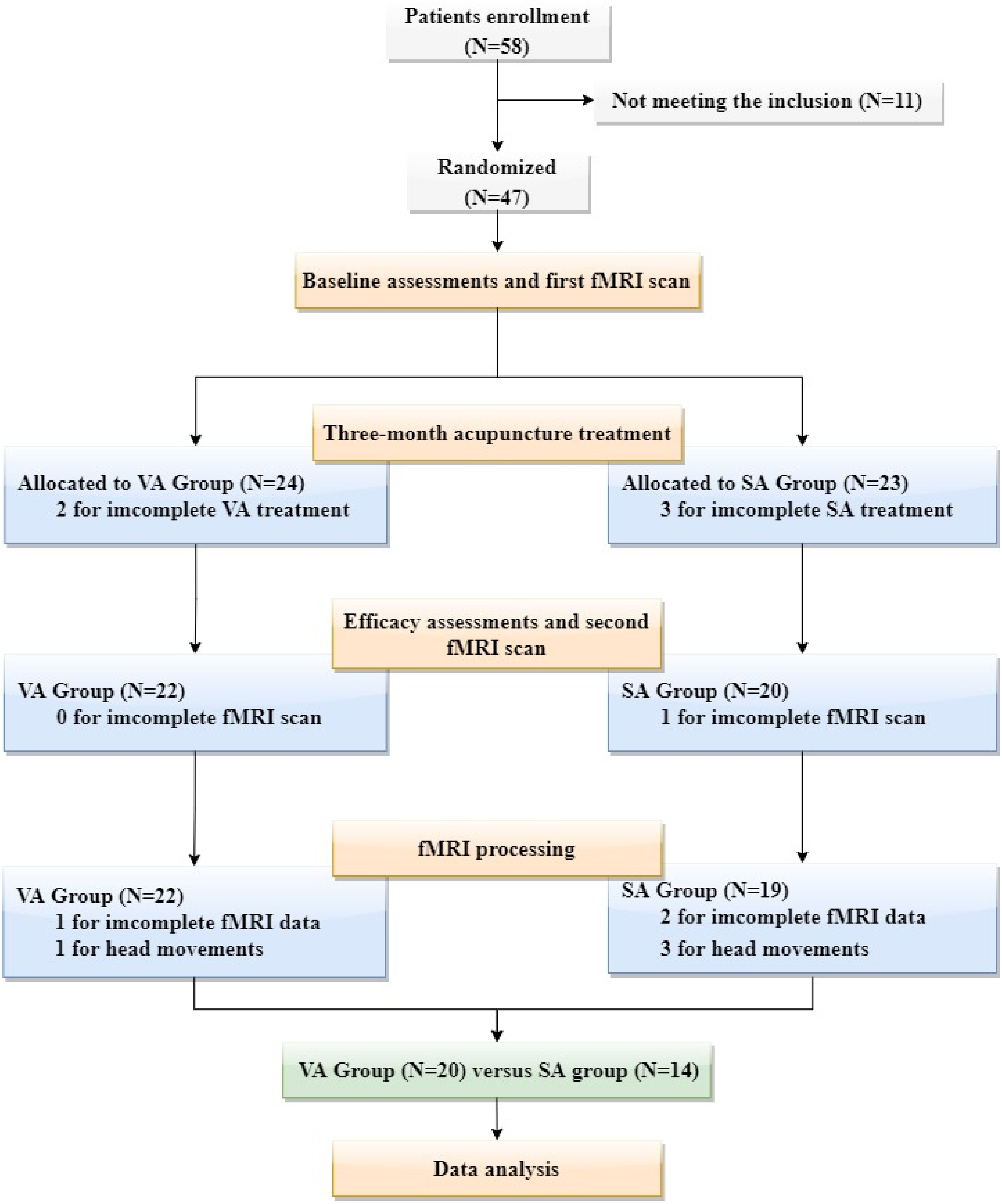

Figure S2. The flow chart of this study. Abbreviations: VA, verum acupuncture; SA, sham acupuncture.

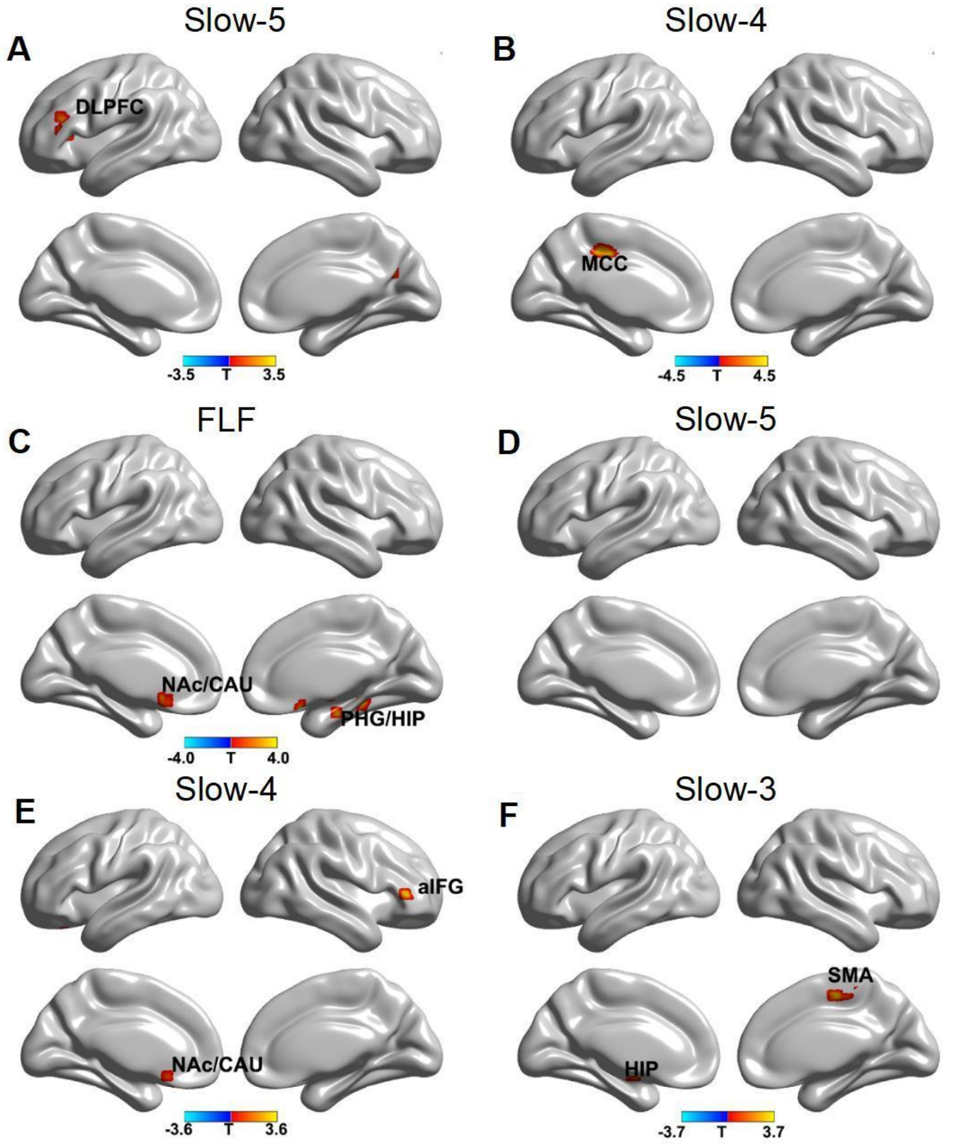

Figure S3. Different distribution patterns of gFCD between verum acupuncture group and sham acupuncture group at multiple frequency bands. A and B showed verum acupuncture increased more gFCD in DLPFC at Slow-5 band and in MCC at Slow-4 band. C, D, E and F showed decreased gFCD distribution difference. C showed verum acupuncture decreased more gFCD in CAU, NAC, HIP/PHG at FLF; E showed verum acupuncture decreased more gFCD in aIFG, CAU, NAC; F showed verum acupuncture decreased more gFCD in SMA, HIP. Abbreviations: gFCD, global functional connectivity density; DLPFC, dorsolateral prefrontal cortex; MCC, middle cingulate cortex; NAC, nucleus accumbens; CAU, caudate nucleus; aIFG, anterior inferior frontal gyrus; SMA, supplementary motor area; HIP, hippocampus.
